# Impact of Cilostazol versus Cilostazol and Selenium Combination on The Healing of Diabetic Foot Ulcer Patients: A Randomized Controlled Trial

**DOI:** 10.1186/s13098-025-02053-4

**Published:** 2026-01-06

**Authors:** Hadeer Eid Eliwa, Lamia M. El Wakeel, Amr A. Mahfouz, Rana Sayed

**Affiliations:** 1 Department of Pharmacy Practice and Clinical Pharmacy, Faculty of Pharmacy, Future University in Egypt, Cairo, Egypt; 2 Department of Clinical Pharmacy, Faculty of Pharmacy, Ain Shams University, Cairo, 11566 Egypt; 3 Department of Internal Medicine, Endocrinology Department, National Institute of Diabetes and Endocrinology, Cairo, Egypt

**Keywords:** Cilostazol, Selenium, Non-ischemic Diabetic Foot Ulcer

## Abstract

**Background:**

Diabetic foot ulcer (DFU) is a debilitating complication of diabetes mellitus (DM). This study aimed to compare the impact of cilostazol alone or with selenium on wound healing in non-ischemic DFU patients**. **

**Methods:**

A randomized controlled trial was conducted on 69 DFU patients. Eligible DFU patients were randomized to; Group1 (n = 23), received standard care only; Group2 (n = 23) received cilostazol plus standard care, Group3 (n = 23) received cilostazol/ selenium, plus standard care, all for 3 months. The Kruskal–Wallis and chi-squared tests were used for comparison among groups.

**Results:**

After 3 months, as compared to controls, cilostazol alone or combined with selenium significantly reduced ulcer size parameters (length, width and depth) with *p* = 0.038, 0.000 and 0.001 respectively. The mean ulcer length, width, and depth in Group1 was reduced by (-1.46 cm, -0.184 cm, and -0.261 cm) respectively. Group2 showed reduction by (-2.704 cm, -1.90 cm and -0.608 cm) respectively and Group3 were reduced by (-3.630 cm, -2.24 cm and -0.50 cm) respectively. There was a complete response to treatment in 30% of group1, 65% of group2, and 43% of group3.

**Conclusion:**

Cilostazol addition to standard wound care significantly improved DFU healing. Addition of selenium to cilostazol had an added benefit to DFU healing on ulcer length and HMMP-9 serum levels.

**Trial registration:**

Clinicaltrials.gov registry (NCT06117436), retrospectively registered on October 19th, 2023.

## Introduction

Diabetic foot ulcer (DFU) is a serious chronic complication of patients with diabetes, resulting in injury and ulcerations within the soft tissues [1, 2]. Foot ulcers occurrence vary between 6% and 16.8% of patients with diabetes [3]. The 5- year mortality rate among DFU patients rises from 50 to 80% following amputation [4]. In DFU patients, delayed wound healing plays a major role [5]. This could be attributed to the prolonged inflammatory response that leads to impairment in keratinocyte migration, collagen formation, fibroblast migration, vascularization, epithelialization, and tissue differentiation [6].

The human matrix metalloproteinase-9 (HMMP-9) is a biochemical marker that has recently been implicated in impaired wound healing of the lower extremity [7, 8], particularly in DFU patients [9, 10] by inhibiting keratinocytes movement to the site of injury and suppressing the synthesis of collagen [5]. Moreover, oxidative stress (OS) induces skin injury, nerve damage, ischemia, and topical infections, which are also involved in the activation of matrix metalloproteinase (MMPs). Increased ROS triggers the activation of Nuclear Factor kappa B (NF-κB), which encourages the expression and up-regulation of MMP-9, a process harmful to diabetic wound healing. The most effective therapeutic approach for treating DFUs involves selective inhibition of the harmful MMP-9 [11].

Cilostazol is a 2‐oxoquinolone derivative that selectively inhibits phosphodiesterase‐3; that down regulates the level of HMMP-9 expression [12], suppresses cyclic adenosine monophosphate (cAMP) degradation, subsequently raising its intracellular level in various body tissue, including endothelium, vascular smooth muscle cells, platelets, adipocytes, and cardio-myocytes [13].Subsequently, cilostazol expresses numerous effects, including antiplatelet‐antithrombotic, anti‐inflammatory, and vasodilatory actions [14–16].

Selenium (Se) is a vital trace element involved in nearly all human physiological processes [17]. Se acts as antioxidant, anti-inflammatory, and anti-viral via acting through at least 25 human seleno-proteins [18, 19]. Selenium as an antioxidant supplement modulates OS biomarkers and eventually positively effects the clinical damage caused by OS [20]. Additionally, it is an essential nutrient for wound healing among patients with DFU, as observed in published studies that have revealed a significant correlation between DFU and selenium levels [21–25]​.

Furthermore, cilostazol and selenium seem to inhibit the expression of HMMP-9 [12, 26].

Hence, the present study aimed to compare the impact of cilostazol alone versus cilostazol and selenium on wound healing, OS and inflammation in non-ischemic DFU patients.

## Material and Methods

### Study Design and Setting

A three-armed, prospective, randomized, controlled, open-label, parallel-group trial was conducted on 69 Egyptian patients over a period of 3-months. This study was conducted on patients with DFUs who attended the National Institute of Diabetes and Endocrinology (NID), Vascular Surgery Outpatients’ clinic. During the period from August 2023 to August 2024. All patients with DFUs who presented to the NID were assessed for eligibility.

### Ethical Approval

The study was performed in accordance with the declaration of Helsinki and its most recent updates in 2024 [27]**.** The study protocol was approved by the ethics committee of the Faculty of Pharmacy, Ain Shams University, (ACUC-FP-ASU) and the National Institute of Diabetes before study-initiation. Moreover, the study was registered at clinicaltrials.gov registry (ID: NCT06117436). All patients were educated about the study protocol, and a written informed consent was signed by each participant without any obligation to withdraw if they want at any time.

### Patients

During the study period, all patients presenting to the NID were assessed for eligibility according to fixed inclusion and exclusion criteria.

#### Eligibility Criteria

Adult patients aged 18 years and older with non-ischemic DFU assessed using ABI (Ankle brachial index) upon recruitment and Wagner’s grade from 2- 4 were considered for inclusion in the study. Excluded patients were those with; Wagner’s grade 1 and 5; poor blood glucose control at the beginning of the study (HbA1c > 12%); or those requiring either graft or percutaneous coronary intervention during the study. Patients allergic to cilostazol or selenium or with contraindications to cilostazol including heart failure, or bleeding disorders; or with a history of other conditions that might interfere with wound healing including cancers, congestive heart failure, end stage renal disease and liver failure were excluded. Similarly, patients presenting with clinical signs of active infection unresponsive to oral antibiotic (edema, fever, redness, discharge, enlarged lymph nodes, or pain); or those with foot wounds due to vascular or dermatological reasons or taking medications that interfere with wound healing (cytotoxic agents, glucocorticoids, and immunosuppressives) upon recruitment were excluded.

#### Randomization

Patients who fulfilled the eligibility criteria were randomly assigned to one of the following three groups; Group 1, control group; Group 2, cilostazol group; or Group 3 cilostazol and selenium group by choosing from closed envelopes that were previously prepared.

Patients in the control group received *standard of care.*

Patients in the cilostazol group received 100 mg of cilostazol once daily [28] in addition to *standard of care***.**

Patients in the cilostazol and selenium group received 100 mg of cilostazol once daily plus, 200 mcg of selenium once daily [29]**,** in addition to *standard of care*. All 3 groups were monitored over a period of 3 months.

***Standard of care*** included wound care (bed rest with elevation, antibiotics, analgesics, dressings and regular consultation) and diabetes management (Insulin and/or oral hypoglycemic medications).

#### Sample Size Calculation

In a study by Razzaghi et al. (2018) [30]; the difference in the change in ulcer depth between magnesium supplementation and placebo groups in patients with diabetic foot ulcer was 0.5 cm with pooled standard deviation of 0.5 cm. Based on these findings, a minimal sample size of 23 subjects in each of group was required at a corrected alpha level of 0.015 and 80% study power (total sample size 69). The Sample size was estimated using PS (Power and Sample Size Program) Version 3.1.2.

### Baseline and Overtime -Assessments

#### Data Collection

Demographic Data (Age, gender, etc.,), smoking status, medical and medication histories, duration of diabetes mellitus and DFUs, were recorded for all groups at baseline.

#### Clinical Evaluation

Wagner-Meggit’s classification was assessed at baseline and monthly thereafter till the end of the study for all groups. Wagner-Meggit’s classification was used to evaluate ulcer grade, wound localization, and presence of infection.

The wound healing response was evaluated by monitoring wound size (ulcer length, width, and depth) reduction (Complete/Partial/lack/Aggravation of healing) at the end of the study (3-month) using a disposable ruler for infection control among the participants as well as the presence of infection.

Also, treatment-related side effects and the occurrence of amputation, mortality and hospital admission for any complications related to DFUs were reported.

#### Laboratory Evaluation

*Glycated hemoglobin (HA1c)* was assessed at baseline and after 3 months in all groups.

*Human matrix metalloproteinase-9 (HMMP-9)* level was evaluated at baseline and at the end of the study. Three ml of venous blood was collected from each patient. Serum was separated by centrifugation, and the supernatant was immediately frozen at −80° C until analysis. Serum levels of HMMP-9 were quantified by enzyme-linked immunosorbent assay (ELISA) kits (Human MMP-9, catalog number: E-EL-H6075, Elabscience®, USA, according to the manufacturer’s instructions.

#### Patient Compliance

Patients’ adherence to medications were recorded. Medication adherence was evaluated by pill count method (a patient is considered non-compliant when the patient fails to take more than 20% of their pills or missed two or more visits).

#### Monitoring Adverse Drug Events

All patients were monitored for the occurrence of adverse drug events through regular visits and follow up in between visits was done by phone calls.

Selenium supplements are generally safe when administered in the recommended doses. However, high doses can lead to side effects that include diarrhea, nausea and fatigue.

The expected side effects from cilostazol were diarrhea, nausea, headache, fatigue, tachycardia, leg cramps, and numbness of tingling.

### Primary and Secondary Outcomes

The primary outcome measure was the effect of cilostazol and selenium on wound healing response.

Secondary outcomes included the effect of cilostazol and selenium on (HMMP-9) level, treatment related side effects, the occurrence of amputation and the mortality or hospital admission for any complications related to DFUs.

### Statistical Analysis

Data management and statistical analyses were performed using the Statistical Package for Social Science version 17 (SPSS Inc., Chicago, IL, USA). The Shapiro–Wilk test was used to ensure the normal distribution of continuous numerical variables. Numerical variables were summarized as the mean ± SD. Categorical data were reported as frequencies and percentages, and the chi-squared test was used to compare categorical variables. Kruskal–Wallis was used for between-group comparisons of non-parametric continuous variables to determine the effects of cilostazol and selenium on ulcer length, width and depth. Simple linear regression was used to evaluate the correlation of HMMP-9 level to continuous variables by the end of the study. *P-values* less than 0.05 (two-tailed) were considered statistically significant. Bonferroni correction was used to adjust the alpha-level for multiple pairwise comparisons during post-hoc analysis (*p-value* < 0.017 was considered significant).

## Results

Out of a total of 206 subjects with DFU screened for eligibility, 125 patients were excluded due to non-eligibility or refusal to join the study. A total of 81 DFU patients were randomized into the three groups; group 1, control group (n = 27), group 2, cilostazol group (n = 27), and group 3, cilostazol plus selenium group (n = 27).

**Fig. 1:**
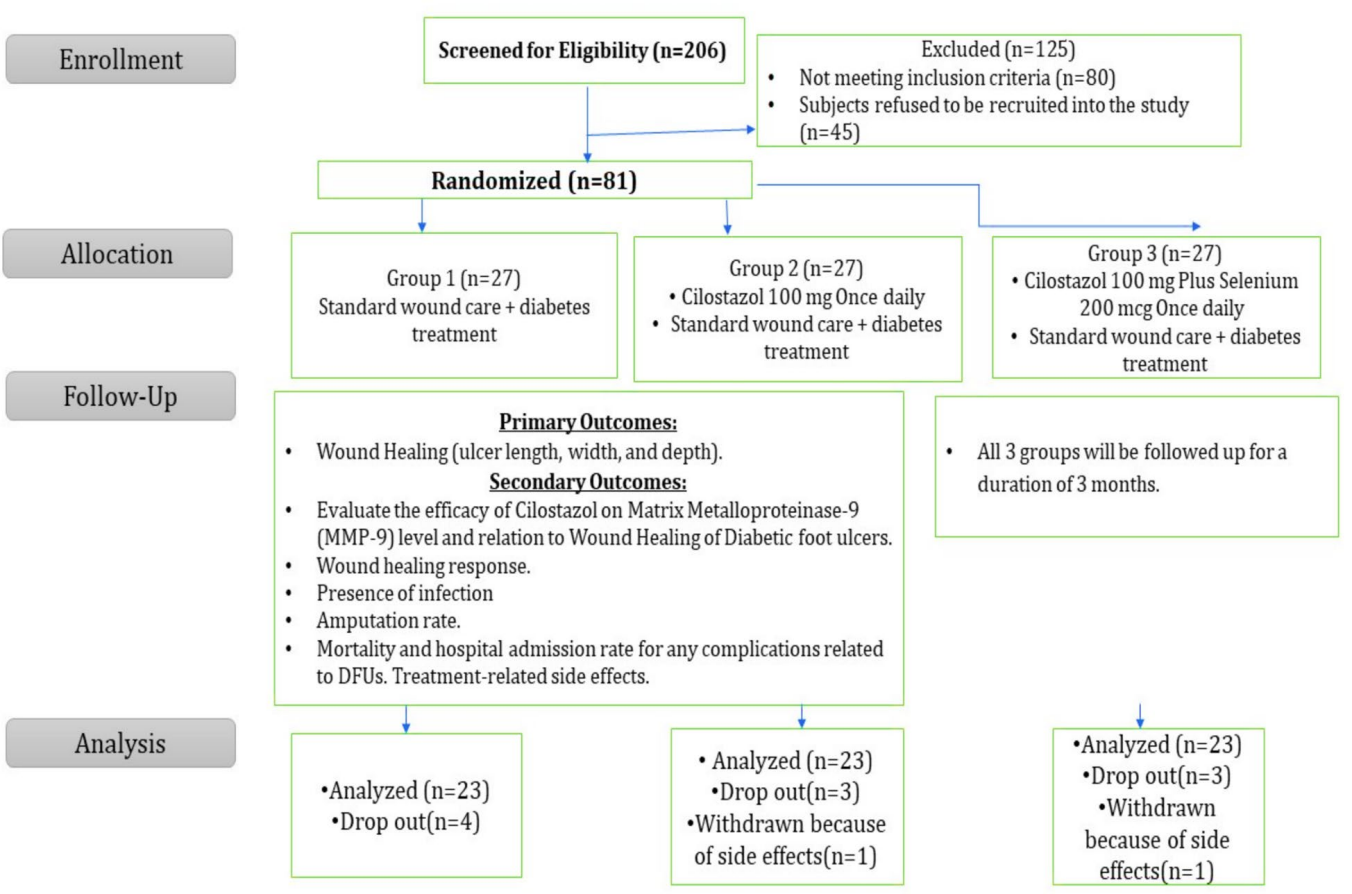
CONSORT flow diagram of the patients recruited

Among the groups, a total of 10 subjects were dropped out for various reasons, ending with 69 subjects with DFU [23 patients per group] completing the trial (Fig. 1).

### Baseline Assessment

The 3 groups were comparable at baseline regarding patient demographics and anthropometrics (age, sex, height and weight), medical history, diabetes status, anti-diabetic regimens, and ulcer characteristics, however, patients in the cilostazol and selenium group had a significantly higher baseline HMMP-9 levels compared to controls (Table 1).

**Table 1:** Demographic data and baseline characteristics of study participants

Characteristics	Total (N = 69)	Group (1) control (N = 23)	Group (2) cilostazol (N = 23)	Group (3) cilostazol + Selenium (N = 23)	*P*-value
Demographic data					
Age (years), mean ± SD	53.203 ± 9.873	49.652 ± 11.183	54.261 ± 8.357	55.696 ± 9.251	0.181 ^a^
Sex; male/female, *n (%)*	55(79.7%)/14(20.3%)	19(82.6%)/4(17.4%)	16(69.6%)/7(30.4%)	20(87%)/3(13%)	0.312 ^b^
HT (cm), mean ± SD	167.928 ± 5.386	169.087 ± 5.435	168.044 ± 5.059	166.652 ± 5.605	0.271 ^a^
WT(Kg), mean ± SD	73.029 ± 10.006	72.565 ± 9.746	74.348 ± 10.259	72.174 ± 10.316	0.804 ^a^
Diabetes characteristics					
Type 1/Type 2 diabetes, *n (%)*	39(56.5%)/30(43.5%)	17(73.9%)/6(26.1%)	12(52.2%)/11 (47.8%)	10(43.5%)/13(56.5%)	0.100^b^
Treatment with insulin/Oral/both,* n (%)*	40(58%)/12(17.4%)/17(24.6%)	16(69.6%)/2(8.7%)/5(21.7%)	14(60.9%)/4 (17.4%)/5(21.7%)	10 (43.5%)/6(26.1%)/ 7(30.4%)	0.424 ^b^
Duration of diabetes (years), mean ± SD	16.797 ± 9.803	18.478 ± 11.285	17.00 ± 9.927	14.913 ± 8.028	0.611 ^a^
Glucose (mmol/l), mean ± SD	139.98 ± 19.289	141.260 ± 18.765	140.826 ± 20.265	137.86 ± 19.496	0.764 ^a^
HbA1c (%), mean ± SD (mmol/mol)	7.8 ± 1.0 62	8.0 ± 0.9 64	7.9 ± 0.9 63	7.5 ± 1.2 59	0.179 ^a^
Ulcer’s baseline characteristics					
Ulcer Length (cm), mean ± SD	4.587 ± 3.521	4.869 ± 3.442	3.565 ± 2.885	5.326 ± 4.049	0.099 ^a^
Ulcer Width (cm), mean ± SD	3.044 ± 1.969	3.022 ± 2.304	2.543 ± 1.499	3.565 ± 1.973	0.174 ^a^
Ulcer Depth (cm), mean ± SD	0.579 ± 0.328	0.631 ± 0.376	0.609 ± 0.425	0.500 ± 0.00	0.253 ^a^
HMMP-9 (ng/l)	69.12 ± 20.25	62.211 ± 20.075(a)	68.43 ± 21.34(a)(b)	76.731 ± 17.28(b)(c)	0.042 ^a*^
Wound Care Compliance; Noncompliant/Compliant,* n (%)*	29(42%)/40(58%)	10(43.5%)/13(56.5%)	8(34.8%)/15(65.2%)	11(47.8%)/12(52.2%)	0.659 ^b^
$Location of DFU,* n (%)*	18(26.1%)/28(40.6%)/7(10.2%/15(21.7%)/1(1.4%)	10(43.5%)/8(34.8%)/2(8.7%)/ 3(13)/0	5(21.7%)/11(47.8%)/1(4.3%)/6(26.2%)/0	3(13.1%)/9(39.1%)/4(17.4%)/6(26.2%)/1(4.3%)	0.263 ^b^
Duration of DFU (Month)	3.9130 ± 2.58098	3.8261 ± 2.54777	3.5000 ± 2.79610	4.4130 ± 2.41516	0.366 ^a^
Concomitant diseases and risk factors, *n (%)*					
Hypertension,* n (%)*	56 (81.2%)/13 (18.8%)	21 (91.3%)/2 (8.7%)	16 (69.6%)/7 (30.4%)	19 (82.6%)/4 (17.4%)	0.165 ^b^
Non-Smoker/Smoker,* n (%)*	47 (68.1%)/22 (31.9%)	13 (56.5%)/10 (43.5%)	18 (78.3%)/5 (21.7%)	16 (69.6%)/7 (30.4%)	0.281 ^b^
Previous amputation (No/Yes),* n (%)*	37 (53.6%)/32 (46.4%)	10 (43.5%)/13 (56.5%)	15(65.2%)/8 (34.8%)	12 (52.2%)/11 (47.8%)	0.330 ^b^

HbA1c, glycated hemoglobin; SD, standard deviation; HMMP-9, Human matrix metalloproteinase 9 $ Location of DFU (Foot dorsal, Sole, Heel, Phalanges &Foot lateral) Statistical tests; ^a^Obtained from The Kruskal-Wallis Test; ^b^Obtained from Pearson chi-square test. *P-value < 0.05. is considered Statistically significant (a, b & c); variables having the same letters are not significantly different & vice versa.

### Primary Outcomes

**Fig. 2:**
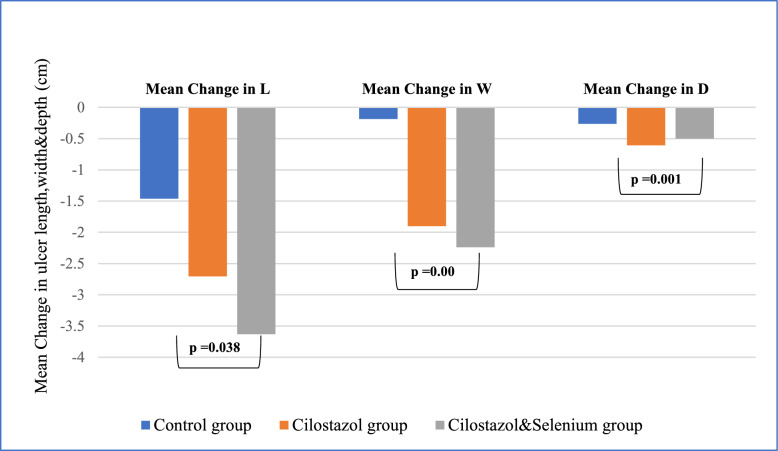
Comparison of the mean change in ulcer length, width and depth between the 3 groups after 3 months. Control group; received standard of care, Cilostazol group; received standard of care plus Cilostazol (100 mg), Cilostazol & selenium; received 100 mg Cilostazol + 200 mcg selenium

After the 3 months treatment, compared to the control group, cilostazol administration resulted in a significant reduction in, width (−1.90 ± 1.226 cm vs −0.184 ± 1.49, *p* = 0.000), and depth (−0.608 ± 0.425 cm vs. −0.261 ± 0.561, *p* = 0.002) but not in ulcer length (−2.704 ± 2.170 cm vs.−1.46 ± 3.25 vs., *p* = 0.068). Similarly, cilostazol and selenium administration, compared to the control group, resulted in a significant reduction in ulcer length (−3.630 ± 2.951 cm vs. −1.46 ± 3.25, *p* = 0.018), width (−2.24 ± 1.857 cm vs. −0.184 ± 1.49, *p* = 0.000), and depth (−0.261 ± 0.561vs.−0.500 ± 0.00 cm, *p* = 0.004) (Table 2) (Fig. 2).

**Table 2:** Comparison of main outcomes at baseline and after the 3 months between the 3 groups

Outcome measures	Group (1) Control (N = 23)			Group (2) cilostazol (N = 23)			Group (3) Cilostazol + selenium (N = 23)			*P*-value
PrimaryOutcomes	Baseline	End of study	Change	Baseline	End of study	Change	Baseline	End of study	Change	
Ulcer length (cm)	4.869 ± 3.441	3.402 ± 3.456	−1.46 ± 3.25 (b)	3.565 ± 2.885	0.860 ± 1.709	−2.704 ± 2.170(a)	5.326 ± 4.049	1.695 ± 2.443	−3.630 ± 2.951(a)	0.038 ^a*^
Ulcer width (cm)	3.021 ± 2.303	2.837 ± 3.044	−0.184 ± 1.49(b)	2.543 ± 1.499	0.643 ± 1.257	−1.90 ± 1.226(a)	3.565 ± 1.973	1.326 ± 1.648	−2.24 ± 1.857(a)	0.000 ^a*^
Ulcer depth (cm)	0.63 ± 0.375	0.369 ± 0.405	−0.261 ± 0.561(b)	0.608 ± 0.425	0.000 ± 0.000	−0.608 ± 0.425(a)	0.500 ± 0.00	0.000 ± 0.000	−0.500 ± 0.00(a)	0.001 ^a*^
Secondary Outcomes										
Wagner Score, n (%)										0.000^b*^
I		10 (43.5%)			21 (91.3%)			23 (100%)		
II& III		13 (56.5%)			2 (8.7%)			0		
HMMP-9(ng/l)	62.211 ± 20.07	66.57 ± 27.96	4.36 ± 28.79	68.43 ± 21.34	67.233 ± 26.90	−1.20 ± 24.13 6	76.731 ± 17.28	65.59 ± 22.58	−11.14 ± 28.16	0.161 ^a^
Wound healing Status, n (%)										0.057 ^b^
Complete		7 (30.43%)			15 (65.2%)			10 (43.5%)		
Partial		4 (17.4%)			8 (34.8%)			13 (56.5%)		
Lack		3 (13%)			0			0		
Aggravation		9 (39.13%)			0			0		
Healing Phase, n (%)										0.002 ^b*^
Epithelialization		9 (39.1%)			18 (78.3%)			10 (43.5%)		
Granulating		9 (39.1%)			5 (21.7%)			13 (56.5%)		
Inflammation		5 (21.8%)			0			0		
Wound Healing Rate, n (%)										
Not Heal		12 (52.2%)			0			0		N/A
After 1 month		0			0			2 (8.7%)		
After 2 months		0			4 (17.4%)			3 (13%)		
After 3 months		11 (47.8%)			19 (82.6%)			18 (78.3%)		
†Infection (No/Yes)		17(73.9%)/ 6 (26.1%)			23 (100%)/0			23 (100%)/0		N/A
Ischemia (No/Yes)		21(91.3%)/ 2 (8.7%)			23 (100%)/0			23 (100%)/0		N/A
Amputation (No/yes)		20 (87%)/ 3 (13%)			23/0			23/0		N/A
$ADE		23(100%)/0			19(82.6%)/ 4(17.4%)			21(91.3%)/2(8.7%)		0.112 ^b^

^$^ADE: Adverse drug event (Not/Dizziness); HMMP-9, Human matrix metalloproteinase 9; †Signs of infection: purulent drainage, heat, erythema, edema, pain and/or loss of function. Statistical tests; a Obtained from The Kruskal–Wallis Test; b Obtained from Pearson chi-square test. *P-value < 0.05 is considered Statistically significant, (a, b & c); variables having the same letters are not significantly different & vice versa

Addition of selenium to cilostazol compared to cilostazol alone did not significantly affect ulcer length (*p* = 0.348), width (*p* = 0.673), or depth (*p* = 0.153).

**Fig. 3:**
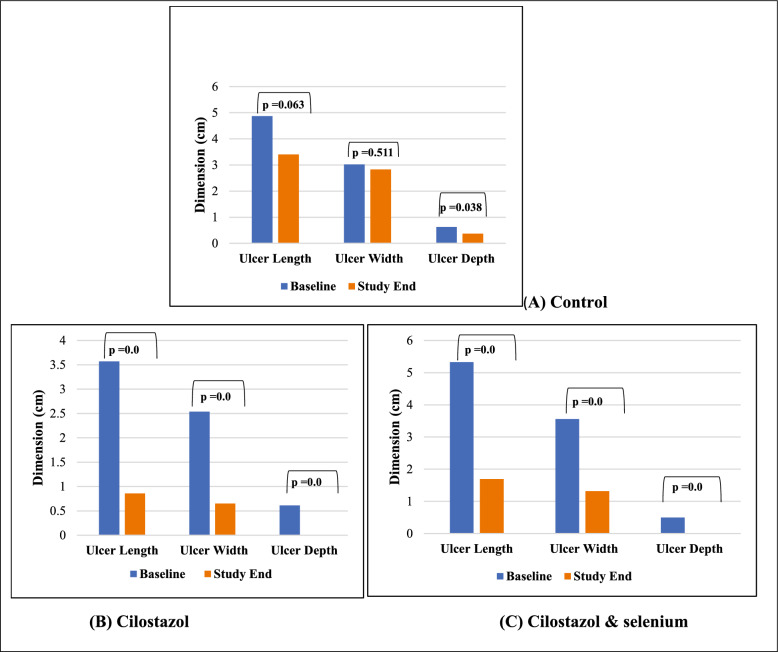
Ulcer Dimensions in control (**A**), cilostazol (**B**) and cilostazol &selenium (**C**) patients from baseline compared to after 3 months

When comparing wound size change from baseline to the end of the study, the control group showed no significant change in ulcer length and width but a significant reduction in ulcer depth from 0.6304 ± 0.375to 0.369 ± 0.405 (*p* = 0.038) was observed. While there was a statistically significant reduction in ulcer length, width, and depth by the end of treatment in both those receiving cilostazol alone or combined with selenium as compared to their baseline. (Fig. 3).

The mean percent ulcer area reductions in cilostazol alone group was 89.8% and cilostazol-selenium combination group was 81.8% by the end of the study.

### Secondary Outcomes

After the 3 months treatment, compared to the control group, taking cilostazol alone or combined with selenium resulted in a significant increase in the number of patients showing complete wound healing (*p* = 0.000), and significant improvement in Wagner score in those receiving cilostazol alone or with selenium from grade II&III to grade I. (Table 2).

At the end of study, Group 1 (control), had 10 (4.3%) Wagner’s grade 1, 8 (34.8%) grade 2 and 5 (21.7%) grade 3 DFU. While, in Group 2 (cilostazol), 21 (91.3%) had Wagner’s grade 1 and 2 (8.6%) had grade 2 DFU. In Group 3(cilostazol plus selenium), 23 (100%) had Wagner’s grade 1.

**Fig. 4:**
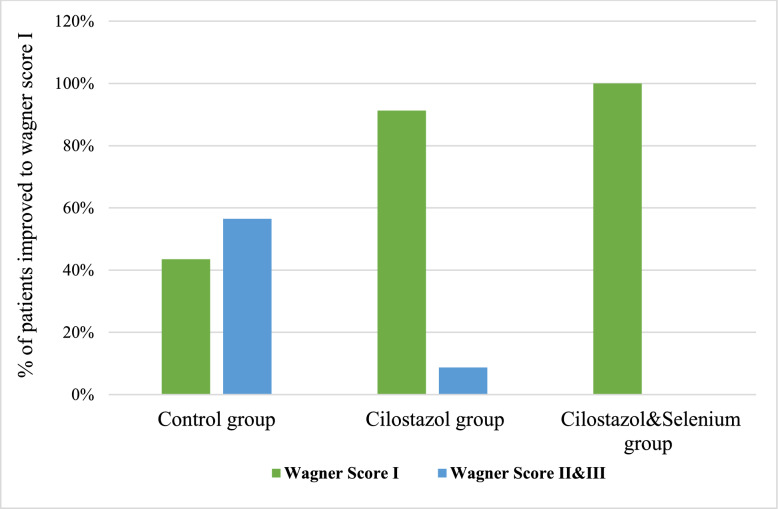
Comparison of Wagner score distribution in the study groups by the end of the study. Control group; received standard of care, Cilostazol group; received standard of care plus Cilostazol (100 mg), Cilostazol & selenium; received 100 mg Cilostazol + 200 mcg selenium

Additionally, the percentage of patients who developed complete ulcer healing showed a higher number in cilostazol (65.20%) compared to cilostazol/selenium (43.5%) compared to controls (30.43%), yet it was not statistically significant (*p* = 0.057) (Fig. 4).

The infection rate was 26% in the control group vs. none in the other two study groups and amputation rate was 13% in control group vs. none in cilostazol and cilostazol + selenium groups.

Also, there was no significant change in HMMP-9 (*p* = 0.161), adverse drug event (*p* = 0.112) and ischemia rate (*p* = 0.127) among the three groups at the end of study (Table 2).

**Fig. 5:**
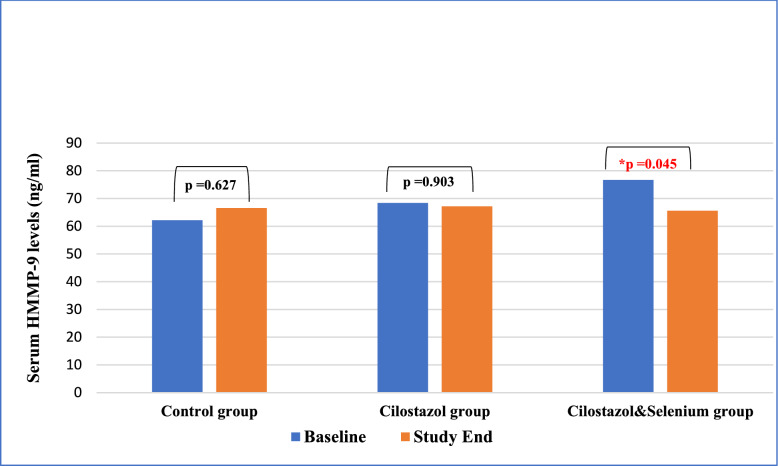
Comparison of HMMP-9 serum levels between the 3 groups at baseline and after 3 months. Control group; received standard of care, Cilostazol group; received standard of care plus Cilostazol (100 mg), Cilostazol & selenium; received 100 mg Cilostazol + 200 mcg selenium

It is worth mentioning that there was statistically significant reduction in the HMMP-9 levels from baseline (76.731 ± 17.28) to (65.59 ± 22.58) (*p* = 0.045) at the end of study in those receiving cilostazol and selenium combination, while there was no significant difference between baseline and end of study HMMP-9 levels in both control and cilostazol group (Fig. 5).

Simple linear regression was used to study the correlation of HMMP-9 serum level by the end of the study to the ulcer dimensions. A weak positive correlation between HMMP-9 and ulcer depth was found (R = 0.264, p-value = 0.029).

None of the patients in the 3 groups were subject to emergency hospital admission or death or any complications related to DFUs.

All patients included were adherent to the medications and those who were not were excluded.

None of the patients in the 3 groups reported any serious medications-related side effects that required interventions. Both the cilostazol and the cilostazol/selenium group reported dizziness that was self-limiting and required no intervention.

## Discussion

The growing rate of DM, which is related to a high occurrence of PVD and a high risk of foot ulceration, has become a serious health, social, and economic burden of worldwide significance. Amputation following diabetic foot ulceration (DFU) negatively affects quality of life and is a leading cause of increased mortality among patients with diabetes [31].

Combined with the purpose of increasing tissue oxygenation and speeding up the healing process, a number of therapies have been studied as supplements to conventional wound care procedures (dressing changes, offloading, and debridement) for patients with DFUs, managing persistent wounds and approaches to endorse healing are important areas of development. Thus, a large group of new interventions is being studied to improve wound healing and consequently diminish amputation and mortality rate among DFU patients [32]**.**

So far to our knowledge, there is an inadequate number of studies evaluating the therapeutic effect of cilostazol or selenium among DFUs patients. Moreover, no studies to date evaluated or compared the efficacy of cilostazol and selenium combination on wound healing among non-ischemic DFU patients versus cilostazol only or versus standard care of DFU therapy. Also, selenium as a mono supplement has not been studied in this patient setting, unlike the published studies that evaluate the effect of selenium in multivitamins that may confound the results.

Previously published studies evaluating the therapeutic effect of cilostazol, and selenium have some limitations. First, their retrospective nature. Second, they used cilostazol as prophylaxis rather than treatment of ischemic DFUs and they did not include the non-ischemic DFU patients. Third, none of the studies evaluated the effect of either cilostazol or selenium on the ulcer size including length, width, and depth. Fourth, ​ no studies were found comparing cilostazol to standard care or any other adjunctive therapy in DFU [29]**.**

In the current study, we evaluated the effects of cilostazol and selenium supplementation on wound healing, and HMMP-9 biomarkers among DFU participants. Our findings demonstrated that cilostazol and selenium supplementation for 3 months among DFU subjects had beneficial effects on ulcer size parameters including (width, length and depth). Similarly, Colak B et al., showed that cilostazol is more effective than aspirin in enhancing wound healing in DFU patients and peripheral artery disease (PAD). The study found that patients receiving cilostazol had a higher rate of complete response to treatment and greater improvement in symptoms compared to those treated with aspirin [28]. Similarly, despite the fact that studies evaluating cilostazol effects on DFU wound healing are lacking, previous evidence suggests that cilostazol promotes healing in various clinical and preclinical settings. For example, studies involving patients with chronic limb-threatening ischemia reported shorter healing times and earlier granulation tissue formation [33, 34]. Similarly, improved one-year healing rates and reduced healing times were observed in patients following bypass surgery for ischemic tissue loss [35]. Additionally, preclinical studies have demonstrated cilostazol’s ability to accelerate bone fracture healing, support tendon repair, and enhance tissue regeneration overall [36].

The current study also demonstrated that the group treated with cilostazol alone achieved a remarkable 65.2% complete ulcer healing, while the control group at 30.4%. However, the cilostazol plus selenium group produced a complete healing of 43.5%. These findings add value to the role of cilostazol in wound healing, particularly in patients with diabetes. Although the study groups showed non statistically significant difference in percentage of patients achieving complete healing by the end of the study with a borderline p-value = 0.057, the cilostazol alone and cilostazol- selenium combination increased percent of complete healing by 34.77% and 13.07%, respectively. These modest percentages can reduce future risk of amputations significantly [37].

The interesting finding of larger ulcer length reduction in cilostazol-selenium combination group compared to cilostazol alone despite lower complete healing rate in the combination group could be explained by antiangiogenic effects of selenium. Zheng et al., conducted a study in a cell model that showed the selenium-chondroitin sulfate nanoparticles suppressed angiogenesis via reduction of vascular endothelial growth factor receptor 2 expression and subsequent inhibition of phosphatidylinositol 3-kinase (PI3K) pathway [38]. In addition, selenium, as an antioxidant, ameliorates hypoxia induced oxidative stress which is considered a main driver of angiogenesis leading to impaired healing process [39]. Moreover, selenium blocked the cAMP/CREB pathway in cancer cells which is known to be involved in gene transcription related to wound healing [40, 41].This might have limited complete wound healing in patients receiving selenium. Studies have shown the microcirculation benefits of cilostazol beyond its ant ischemic effects including enhanced perfusion and improved endothelial function [42, 43]. These potential microcirculatory effects might help improve wound healing of non-ischemic DFU.

In comparison to pentoxifylline, a phosphodiesterase inhibitor with microcirculation effects similar to cilostazol, our study documented that the percent of DFU patients (Wagner’s grade 2–4) showing recovery (partial or complete wound healing) was higher in those receiving cilostazol alone (100%) or cilostazol-selenium combination (100%) while Rewale et al., reported 86.66% of DFU patients (Wagner’s grade 1–2) showing signs of recovery with pentoxifylline [44]. This difference could be attributed to the fact that pentoxifylline was administered only for one month which might have limited its efficacy.

In the current study, the mean percent ulcer area reductions in cilostazol and cilostazol-selenium combination groups were 89.8% and 81.8%, respectively. This was better than percent ulcer area reduction reported in patients treated with topical platelet-derived growth factor (61.7%) [45]. This emphasize the importance of systemic therapy compared to topically administered adjuvants.

Comparing our results with current literature, a retrospective analysis reported that 36.6% of patients showed healing without requiring surgical interventions or revascularization [46]. This rate is relatively lower than our findings, which could be attributed to several causes, including differences in ulcer characteristics, the timing and method of intervention and the patient demographics.

Our study findings provide compelling evidence on the efficacy of combining cilostazol and selenium in improving the healing of DFUs. After 3 months of treatment, the selenium/cilostazol group demonstrated a significant reduction in ulcer size compared to the control group but no statistically significant difference when compared to the cilostazol only group.

Comparing our results to those from studies investigating other antioxidant supplementation, such as magnesium, zinc, and omega-3 fatty acids. A study that investigated the efficacy of magnesium supplementation, significant reductions in ulcer length (− 1.8 cm), width (− 1.6 cm), and depth (− 0.8 cm) were reported after 12 weeks [30]. While magnesium showed beneficial effects, our treatment showed higher reduction in all measured ulcer size parameters.

Similarly, the findings of investigating zinc supplementation revealed reductions in ulcer length (− 1.5 cm), width (− 1.4 cm), and depth (− 0.8 cm), also with statistically significant results (P < 0.05) [47]. The current study’s intervention demonstrated a markedly greater impact on ulcer size.

Moreover, considering the effects of omega-3 fatty acids, which revealed decreases in ulcer length (− 2.0 cm), width (− 1.8 cm), and depth (− 0.8 cm) with statistical significance (P < 0.05)[48], it becomes clear that while omega-3 s are beneficial, they still fall short when compared to our combination treatment.

It is worth noting that in the current study, observed changes in ulcer size in the cilostazol and selenium supplementation groups compared to control group were statistically significant. These findings are consistent with previous studies demonstrating the efficacy of cilostazol in improving circulation in PAD and the role of selenium in enhancing wound healing through antioxidant mechanisms [22, 28, 49].

The human matrix metaloprotinases-9 (HMMP-9) has recently been implicated in impaired wound healing of the lower extremity particularly in DFU patients and hence could be a potential target marker to guide DFU outcomes. Yet, the current study failed to show a statistically significant difference in the HMMP-9 serum levels between the cilostazol group and the cilostazol plus selenium group compared to the control group. However, it is noteworthy that there was a statistically significant decrease in HMMP-9 levels within the cilostazol plus selenium group from baseline to the end of the study while there was no significant difference in the other study groups. These findings suggest that while cilostazol alone may not have a strong impact on HMMP-9 levels in the short term, the addition of selenium might enhance its efficiency in decreasing these levels over time due to selenium potentially increasing the expression of tissue inhibitor metalloproteinase (TIMP)−1 [50]. The balance of both HMMP-9 and TIMP-1 is a predictor of keratinocyte migration and extracellular matrix modulation essential for wound healing [51]. This modulation of collagen deposition might have affected tensile strength and longitudinal wound contraction which might explain the preferential significant reduction in ulcer length in those receiving selenium compared to its effect on ulcer width and depth. In line with the potential beneficial effect of selenium in the current study, a preclinical model of cisplatin-induced nephrotoxicity demonstrated that selenium supplementation effectively reduced elevated serum levels of MMP-9 [52]. While cilostazol is generally known to suppress MMP-9 expression and activity, contributing to its anti-inflammatory and vascular protective effects, however, its impact on MMP-9 levels may vary depending on the disease context. Several clinical studies have reported that cilostazol significantly reduces serum MMP-9 levels in various conditions, including silent brain infarcts [53], atherosclerosis [54], unstable angina, and myocardial infarction [55].

In contrast, Franciscis et al. [49], reported that cilostazol significantly reduced HMMP-9 plasma levels. The discrepancy between our study findings and those of Franciscis et al. could be attributed to the difference in the patient population studied, as well as its significant contribution to the difference in the dosing regimens used between the 2 studies (100 mg twice daily) as compared to (100 mg once daily) in the current study. Moreover, it is worth noting that in the current study, the cilostazol and cilostazol/selenium group had significantly worse HMMP-9 plasma levels compared to the control group, which could explain the reason behind the non-significance between the 3 groups in the HMMP-9 levels by the end of the study despite the fact that the cilostazol/selenium group showed a significant decline in HMMP-9 levels when comparing baseline vs end of study levels (p-value = 0.045).

Collectively, it can be concluded that despite the fact that selenium/cilostazol addition showed a similar healing to that of cilostazol alone, selenium addition showed a more favorable effect on HMMP-9 levels as well as a more pronounced impact on ulcer length which could be attributed to the heterogeneity of the healing process that could be affected by ulcer location, blood supply and individual healing capacities.

Hence, Cilostazol and selenium are promising adjunct therapies for DFUs management. Their effects in improving blood flow and reducing OS make them a valuable option for enhancing DFU healing. Both cilostazol and selenium were tolerable, and no patients developed adverse events that required drug discontinuation or interventions.

## Limitations and Recommendations

The moderately small sample size in our study, although the study being powered to the primary outcome, might have affected detection of clinically significant differences in secondary outcomes. In addition, the open-label design might have introduced a risk of measurement bias despite employed techniques to reduce this bias via including HMMP-9 as an objective biochemical marker and ensuring that the investigator responsible for ulcer measurement was blinded to treatment allocation. The imbalance in baseline HMMP-9 levels being higher in the combination group compared to the control group might have influenced the observed changes. Furthermore, the study duration was short. Future studies should investigate the additive effects of cilostazol and selenium at different doses over longer periods of time and on larger sample sizes. In addition, lack of studies on the potential role of selenium alone in non-ischemic DFU healing has limited the discussion of the effect of combining cilostazol and selenium versus selenium alone. We recommend future studies on the effect of selenium supplementation on DFU healing.

## Conclusion

In conclusion, our study demonstrated that cilostazol alone or combined with selenium supplementation are promising tolerable adjuvant treatments for the management of DFU.

## Data Availability

The datasets generated during and/or analyzed during the current study are available from the corresponding author on reasonable request.
